# Metabolic syndrome and inflammatory biomarkers: a community-based cross-sectional study at the Framingham Heart Study

**DOI:** 10.1186/1758-5996-4-28

**Published:** 2012-06-20

**Authors:** Dhayana Dallmeier, Martin G Larson, Ramachandran S Vasan, John F Keaney, Joao D Fontes, James B Meigs, Caroline S Fox, Emelia J Benjamin

**Affiliations:** 1 General Internal Medicine Division, Boston University School of Medicine, Boston, USA; 2 National Heart, Lung, and Blood Institute’s and Boston University’s Framingham Heart Study, Framingham, USA; 3 Cardiology, Whitaker Cardiovascular Institute, Boston University School of Medicine, Boston, USA; 4 Preventive Medicine Divisions, Whitaker Cardiovascular Institute, Boston University School of Medicine, Boston, USA; 5 Biostatistics Department, Boston University School of Public Health, Boston, USA; 6 Epidemiology Department, Boston University School of Public Health, Boston, USA; 7 Department of Medicine, Harvard Medical School, Boston, USA; 8 Department of Mathematics and Statistics, Boston University, Boston, USA; 9 Cardiovascular Medicine, University of Massachusetts Medical School, Worcester, USA; 10 Department of Endocrinology, Diabetes, and Metabolism, Brigham and Women’s Hospital and Harvard Medical School, Boston, USA

**Keywords:** Metabolic syndrome, Inflammatory biomarkers, Body mass index, Insulin resistance

## Abstract

**Background:**

Prior studies reported conflicting findings on the association between metabolic syndrome and inflammatory biomarkers. We tested the cross-sectional associations between metabolic syndrome and nine inflammatory markers.

**Methods:**

We measured C-reactive protein, CD40 ligand, interleukin-6, intercellular adhesion molecule-1, monocyte chemoattractant protein-1, osteoprotegerin, P-selectin, tumor necrosis factor-alpha, and tumor necrosis factor receptor-2 in 2570 Framingham Offspring Study participants free of diabetes and cardiovascular disease at examination 7. Metabolic syndrome was defined by National Cholesterol Education Program criteria. We performed multivariable linear regressions for each biomarker with metabolic syndrome as the exposure adjusting for age, sex, smoking, aspirin use, and hormone replacement. We subsequently added to the models components of the metabolic syndrome as continuous traits plus lipid lowering and hypertension treatments. We considered *P* < 0.05 as statistically significant.

**Results:**

Metabolic syndrome was present in 984 (38%) participants and was statistically significantly associated with each biomarker (all *P <* 0.02) except osteoprotegerin. After adjusting for its component variables, the metabolic syndrome was associated only with P-selectin (1.06 fold higher in metabolic syndrome, 95% CI 1.02, 1.10, p = 0.005).

**Conclusions:**

Metabolic syndrome was associated with multiple inflammatory biomarkers. However, adjusting for each of its components eliminated the association with most inflammatory markers, except P-selectin. Our results suggest that the relation between metabolic syndrome and inflammation is largely accounted for by its components.

## Background

Obesity, insulin resistance and type 2 diabetes mellitus have been characterized as chronic “inflammatory” states that are associated with abnormal concentrations of cytokines, acute-phase reactants and other inflammatory signaling markers [1-5]. An association between metabolic syndrome and an elevated risk of developing diabetes mellitus and cardiovascular disease also has been described [6-8]. In addition, consistent associations between elevated mean C-reactive protein (CRP) concentrations and body weight and metabolic syndrome have been demonstrated [9-12].

According to the most recent World Health Organization expert consultation with respect to metabolic syndrome, future research should focus on further elucidation of common metabolic pathways underlying the development of diabetes and cardiovascular diseases [13]. Furthermore, the shift in mean body mass index (BMI) towards higher levels in all age and sex groups in the US [14] and to an increased prevalence of metabolic syndrome [15], has contributed to growing interest in evaluating the association between metabolic syndrome and inflammatory biomarkers [12].

Prior studies have examined the association of metabolic syndrome to one or a few inflammatory biomarkers, in modest-sized cohorts reporting conflicting findings [2,9,10,12,16]. The available literature has been inconclusive as to whether metabolic syndrome is a better predictor of the development of diabetes mellitus and/or cardiovascular disease when compared to its individual factors. Some experts have suggested that the risk associated with the syndrome is explained by the presence of its components [17-19].

We evaluated the association between metabolic syndrome and a panel of nine inflammatory biomarkers in the community-based Framingham Heart Study. The nine biomarkers were chosen to represent different phases and processes in inflammatory pathways as detailed elsewhere [20,21]. Briefly, CRP is a nonspecific acute phase reactant; interleukin-6, tumor necrosis factor alpha, and tumor necrosis factor receptor 2 represent cytokines; monocyte chemoattractant protein-1 contributes to leukocyte recruitment; P-selectin is responsible for leukocyte tethering; CD40 ligand contributes to cellular immunity; intercellular adhesion molecule-1 contributes to leukocyte adhesion; and osteoprotegerin is a member of the tumor necrosis factor receptor family. We tested the hypothesis that the relation between metabolic syndrome and inflammation is accounted for by the components of the metabolic syndrome. In the presence of heterogeneity in the metabolic state among the different BMI categories [22], we hypothesized that knowledge of inflammatory biomarkers might help to understand differences between ‘metabolically healthy but obese’ and ‘metabolically obese but normal weight’ individuals.

## Methods

The present cross-sectional study was conducted in the Framingham Heart Study, a community-based observational epidemiological project. The design and selection criteria of the Framingham Offspring study have been described [23]. Individuals (n = 3539) who participated at the 7^th^ examination cycle (1998 to 2001) were evaluated in this study. Participants were excluded for the following reasons (in order): off-site examination (n = 206); prevalent diabetes, defined as fasting plasma glucose ≥126 mg/dL or use of insulin or oral hypoglycemic agents (n = 449); prevalent cardiovascular disease, defined as a history of angina pectoris, coronary insufficiency, myocardial infarction, heart failure, transient ischemic attack, stroke, or intermittent claudication, determined by a panel of three physicians (n = 305); missing information regarding metabolic syndrome traits or insulin treatment (n = 9). The study protocol was reviewed and approved by the Institutional Review Board of Boston University Medical Center; all participants gave written consent.

### Covariate assessment

The covariates were defined at examination cycle 7 through assessment of questionnaires, physicals and laboratory tests. Current smoking status was classified by self-report of cigarette smoking during the year prior to examination. Resting blood pressure was measured in a seated position by the physician using a mercury column sphygmomanometer; blood pressure represented the average of two readings. Hypertension was defined as systolic blood pressure ≥140 mmHg or diastolic blood pressure ≥90 mmHg or use of antihypertensive medications. Waist circumference in centimeters was measured by trained technicians at the umbilicus level according to a standard protocol. BMI was defined as the individual’s body weight divided by the height squared, expressed in kg/m^2^. Obesity was defined as BMI ≥30 kg/m^2^. Lipid profile, plasma glucose and insulin levels were measured from morning fasting blood samples using standardized assays. We calculated homeostasis model assessment insulin resistance index (HOMA-IR) by applying the formula [(fasting insulin)(fasting glucose)]/22.5. Insulin resistance was considered present if the insulin resistance index (HOMA-IR) was ≥75^th^ percentile. Aspirin use was defined as 3 or more doses per week.

### Measurement of inflammatory and oxidative stress marker concentrations

Fasting samples were frozen at −80° Celsius until testing. Serum concentrations were measured for CRP, interleukin-6, intercellular adhesion molecule-1, monocyte chemoattractant protein-1. Plasma concentrations were estimated for CD40 ligand, osteoprotegerin, P-selectin, tumor necrosis factor-alpha, and tumor necrosis factor receptor 2. CRP was measured through high sensitivity Dade Behring BN100 nephelometer. Other biomarkers were assessed by enzyme linked immunoassay; all intra-assay coefficients of variation were ≤9.1%. Details regarding marker selection and measurements have been reported [24].

### Definition of metabolic syndrome

Metabolic syndrome was defined according to the National Cholesterol Education Program Adult Treatment Panel III guidelines [5] as elucidated in Table 1. Metabolically healthy but obese individuals were defined as participants with BMI ≥30 kg/m^2^, but without the metabolic syndrome. Metabolically obese but normal weight individuals were identified as those with the metabolic syndrome and BMI <25 kg/m^2^.

**Table 1:** Participant characteristics

		**Metabolic Syndrome**	
		**No (n = 1586)**	**Yes (n = 984)**
Age, years		58 ± 9	62 ± 9
Women, %		60	52
Body mass index, kg/m^2^		25.9 ± 4.2	30.5 ± 5.0
Mean waist, cm		93 ± 12	106 ± 11
Systolic blood pressure, mmHg		120 ± 17	133 ± 18
		22	64
Hypertension treatment, %		15	44
		14	11
Triglycerides, mg/dl		101 ± 53	171 ± 91
Total/HDL cholesterol		3.5 ± 1.0	4.6 ± 1.3
Lipid treatment, %		5	27
Aspirin use, %		22	29
Hormone replacement therapy, %		20	16
Metabolic syndrome components, % (n)			
Elevated waist circumference	≥102 cm in men, ≥88 cm in women	41 (655)	88 (862)
Elevated triglycerides	≥150 mg/dL	16 (248)	69 (679)
Low HDL	<40 mg/dL in men, <50 mg/dL in women	11 (169)	53 (522)
High blood pressure	Systolic BP ≥130, diastolic BP ≥85 mmHg or treatment	32 (502)	79 (778)
Hyperglycemia	Fasting glucose ≥100 and <126 mg/dL	17 (273)	67 (661)

Data are mean ± standard deviation and % (n). BP denotes blood pressure.

### Statistical analyses

The inflammatory markers concentrations showed skewed distributions and were natural log-transformed before further analysis. We performed multivariable linear regression with biomarkers as dependent variables adjusting for age, sex, smoking, aspirin use, and hormone replacement therapy. In model 1, metabolic syndrome was the key exposure. In model 2, we added adjustment for metabolic syndrome components as continuous traits while adjusting for age, sex, smoking, aspirin use, hormone replacement, lipid lowering treatment and hypertension therapy. In model 3, we examined inflammatory biomarkers with interaction between metabolic syndrome and BMI: normal weight was defined as BMI <25 kg/m^2^, overweight as BMI 25–29.99 kg/m^2^ and obese as BMI ≥30 kg/m^2^. With model 4, we analyzed inflammatory biomarkers with the interaction between metabolic syndrome and insulin resistance. We estimated fold increment (and 95% CI) comparing adjusted biomarker levels in groups with and without metabolic syndrome. We considered *P* <0.05 as statistically significant. The statistical analysis was performed with SAS version 8.1 (SAS Institute, Cary, NC, USA).

### Secondary analyses

In secondary analyses, tests for sex interaction with metabolic syndrome were performed for the inflammatory biomarkers, adjusting for age, smoking, aspirin and hormone replacement therapy using a significance level of *P* < 0.01.

## Results and discussion

Table 1 shows clinical characteristics of the 2570 participants. The study had 984 (38%) participants with (mean age 62 ± 9 years, 52% women) compared to 1586 participants without (mean age 58 ± 9 years, 60% women) the metabolic syndrome.

### Regression model for the individual markers

Multiple linear regression models demonstrated highly statistically significant associations (*P* <0.0001) between prevalent metabolic syndrome and CRP, interleukin-6, intercellular adhesion molecule-1, P-selectin, tumor necrosis factor-alpha, and tumor necrosis factor receptor-2,after adjusting for age, sex, smoke, aspirin and hormone replacement therapy. CD40 ligand and monocyte chemoattractant-1 also were nominally associated (P ≤ 0.02), but osteoprotegerin was not associated with the metabolic syndrome. Except for osteoprotegerin, inflammatory biomarkers showed higher mean concentrations in participants with versus without metabolic syndrome (Table 2, model 1).

**Table 2:** Fold increments for inflammatory biomarkers comparing those with metabolic syndrome (n = 984) versus those without the metabolic syndrome (n = 1586)

**Biomarker**		**Model 1^†^**		**Model 2 - with additional adjustment for the components of the metabolic syndrome^‡^**		
	**Sample Size**	**Estimate (95% CI)**	**P-value**	**Sample Size**	**Estimate (95% CI)**	**P-value**
C-reactive protein	2555	1.82 (1.67, 1.97)	<0.0001	2551	1.07 (0.96, 1.19)	0.23
CD40Ligand	2559	0.89 (0.80, 0.98)	0.02	2555	0.89 (0.77, 1.02)	0.10
Intercellular adhesion molecule-1	2557	1.05 (1.03, 1.07)	<0.0001	2553	1.00 (0.97, 1.03)	0.99
Interleukin-6	2553	1.29 (1.22, 1.36)	<0.0001	2549	1.02 (0.94, 1.10)	0.67
Monocyte chemoattractant −1	2517	1.03 (1.01, 1.06)	0.01	2513	0.98 (0.95, 1.02)	0.34
Osteoprotegerin	2555	1.00 (0.98, 1.02)	0.92	2551	0.99 (0.96, 1.02)	0.65
P-selectin	2558	1.11 (1.08, 1.15)	<0.0001	2554	1.06 (1.02, 1.10)	0.005
Tumor necrosis factor-alpha	1948	1.10 (1.05, 1.15)	<0.0001	1945	0.99 (0.93, 1.06)	0.84
Tumor necrosis factor receptor 2	2505	1.09 (1.06, 1.11)	<0.0001	2501	1.00 (0.97, 1.03)	0.91

† Adjusted for age, sex, smoking, aspirin use, hormone replacement therapy.

‡ Adjusted for age, sex, smoking, aspirin use, hormone replacement therapy, lipid lowering medications and hypertension treatment, as well as for the components of the metabolic syndrome: waist circumference, triglycerides level, HDL level, systolic and diastolic blood pressure and anti-hypertensive therapy, and fasting blood glucose.

### Accounting for metabolic syndrome components

Table 2 (model 2) shows the relation of the metabolic syndrome to inflammatory biomarkers after adjusting for the components of the metabolic syndrome. In that setting, metabolic syndrome remained significantly associated only with P-selectin (*P* = 0.005). Subjects with metabolic syndrome had a 1.06 fold (i.e., 6%; (95% CI 1.02, 1.10, P = 0.005)) increase of P-selectin compared to those without metabolic syndrome.

### Interaction between metabolic syndrome and BMI

Only 10% (n = 101) of individuals with metabolic syndrome were normal weight, 43% (n = 421) were overweight and 47% (n = 462) were obese (Additional file 1 Table S1). In participants without metabolic syndrome, the distribution was shifted to lower mean BMI (45% normal weight, 41% overweight and 14% obese). Among normal weight participants, 12% had the metabolic syndrome. Obesity was observed in 680 participants; 32% (n = 218) were metabolically healthy but obese. Among normal weight individuals, the metabolic syndrome was associated with higher mean concentrations of the following biomarkers: CRP, intercellular adhesion molecule-1, interleukin-6, P-selectin, tumor necrosis factor-alpha and tumor necrosis factor receptor 2 when compared to healthy normal weight individuals (Additional file 1 Table S1).

**Figure 1:**
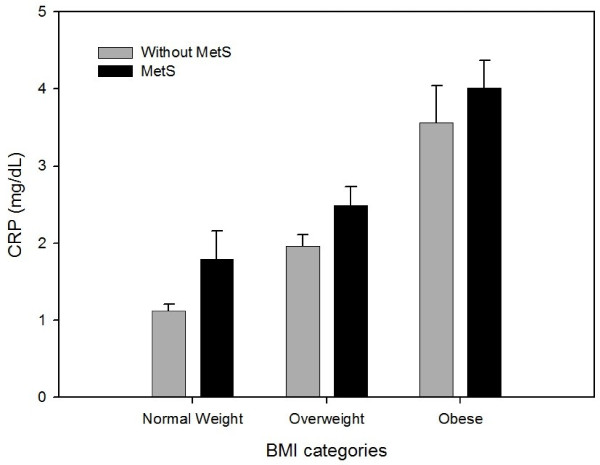
**Geometric mean concentrations of C-reactive protein (CRP) by BMI category with/without metabolic syndrome (MetS) obtained from the multivariable-adjusted regression model with natural log(CRP) as dependent variable adjusting for age, sex, smoking, aspirin use and hormone replacement therapy.** Whiskers extend to upper limits of two-sided 95% confidence intervals.

The interaction between metabolic syndrome and BMI category was statistically significant for CRP (*P* = 0.02). The proportional increase in CRP, comparing those with to those without metabolic syndrome, decreased across BMI categories (Figure 1). The presence of metabolic syndrome was associated with a 1.60 fold (i.e. 60%; (95% CI 1.31, 1.95 fold)) increase of the mean CRP concentration than would be anticipated in normal weight individuals, with a 1.27 fold (i.e. 27%; (95% CI 1.13, 1.43 fold)) increase among overweight subjects, but with a non-significant 1.13 fold (i.e. 13%; (95% CI 0.96, 1.31 fold)) increment in obese individuals (Additional file 1 Table S1).

### Interaction between metabolic syndrome and insulin resistance

In individuals with metabolic syndrome 49 % had insulin resistance, compared to 10 % in those without metabolic syndrome The interaction between metabolic syndrome and insulin resistance was statistically significant only for CRP (*P* = 0.008). Metabolic syndrome without insulin resistance was associated with a 1.67 fold (i.e. 67%; (95% CI 1.50, 1.86 fold)) increase of mean CRP levels compared to those individuals without metabolic syndrome and insulin resistance. Among those with insulin resistance, metabolic syndrome was associated with a 25% increment (1.25 fold (95% CI 1.04, 1.51 fold)) of mean CRP levels compared to those without the metabolic syndrome (Additional file 2 Table S2). When evaluating participants without metabolic syndrome, those with insulin resistance had statistically significant higher mean concentrations of CRP compared to those without insulin resistance, and their levels were similar to those individuals with metabolic syndrome but without insulin resistance. Mean CRP levels were highest in individuals with both conditions (Additional file 3 Figure S1).

### Secondary analyses

The interaction of metabolic syndrome and sex was statistically significant for CRP (*P* < 0.0001) and tumor necrosis factor receptor 2 (*P* = 0.002). Among women, we observed in the presence of metabolic syndrome a statistically significant 2.17 fold increment of mean CRP levels (95% CI 1.95, 2.43), whereas in men the observed increment was 1.47 fold (95% CI 1.30, 1.65). Regarding tumor necrosis factor receptor 2, women with metabolic syndrome had higher mean concentrations (fold increment 1.12, 95% CI 1.09, 1.15) compared to those without; among men the corresponding increment was 1.05 fold (95% CI 1.01, 1.08) (Additional file 4 Table S3).

We observed a significant association between the metabolic syndrome and all inflammatory biomarkers except osteoprotegerin, which is consistent with the hypothesis that the metabolic syndrome is accompanied by an inflammatory state. We report an interaction between BMI and metabolic syndrome for CRP; in individuals with obesity the presence of the metabolic syndrome did not appear to be associated with additional elevation in mean CRP concentrations. We also detected a significant interaction for CRP in relation to the metabolic syndrome and insulin resistance. Among those without the metabolic syndrome, the presence of insulin resistance was associated with higher mean concentrations of CRP. When evaluating metabolically obese but normal weight individuals, we observed higher mean concentrations of CRP, intercellular adhesion molecule-1, interleukin 6, P-selectin, tumor necrosis factor-alpha and tumor necrosis receptor 2 compared to healthy normal weight individuals. Our results reinforce the concept that the metabolic syndrome even in the absence of obesity is associated with an inflammatory state. Finally, we demonstrated that adjusting for all the components of the metabolic syndrome attenuated the association between the metabolic syndrome with all biomarkers, except P-selectin.

The association between metabolic syndrome and some of the inflammatory biomarkers has been examined in the past [2,9,10,12,16]. The current literature provides evidence of elevated levels of CRP, tumor necrosis factor alpha, interleukin 6 in individuals with central fat when compared to those with normal fat distribution [25,26]. In the same cohort at the Framingham Heart Study, we demonstrated that tumor necrosis factor alpha and tumor necrosis factor alpha receptor 2 remained associated with insulin resistance after adjusting for central obesity, adiponectin and resistin [27].

Consistent with our results increased levels of P-selectin have been described among individuals with as compared to without the metabolic syndrome [28,29]. An increased expression of cell adhesion molecules such as intercellular adhesion molecule-1 and P-selectin have also been associated in a smaller cohort with increased waist circumference, low HDL cholesterol and elevated fasting glucose [16]. P-selectin is known to be involved in the attachment of circulating leukocytes to the vascular endothelium, contributing to the early development of atherosclerotic lesions, even before a metabolic disorder would be detected. It is expressed on activated platelets as well as by endothelial cells. The secretion of P-selectin can be induced through atherogenic factors such as oxidized LDL. Nevertheless the association between P-selectin and the metabolic syndrome after adjusting for its components although statistically significant, warrants cautious interpretation. We may have increased the chance of introducing false positive results by multiple testing. The clinical significance of the reported association merits further study.

### Clinical implications and future directions

We recognize the controversy surrounding the use of the metabolic syndrome as a diagnostic or management tool, understanding its role as a pre-morbid condition rather than a clinical diagnosis [13]. In this regard it has been estimated that about one fifth of the US population fulfills the criteria of the metabolic syndrome [15]. Further studies evaluating the role of the inflammatory biomarkers among metabolically healthy but obese and metabolically obese but normal weight individuals compared to their counterparts are needed in order to enhance our understanding regarding the pathophysiology behind the observed clustering of abnormal metabolic traits. We acknowledge that the clinical significance of our findings is uncertain. Further work should investigate whether inflammatory markers will prove useful in the early identification of individuals at risk for the development of the metabolic traits, and whether such risk stratification will be associated with the ability to reduce or delay the incidence of associated morbidity and mortality.

### Strengths and limitations

Given our cross-sectional observational design, our study cannot prove causality. It is possible that metabolic features lead to inflammation, or that inflammation predisposes to the development of metabolic perturbations, or that a complex feedback loop exists wherein each fuels the development and progression of the other. Alternatively both inflammation and metabolic traits may be both related to additional untested features.

Of the various available definitions for the metabolic syndrome, we used the National Cholesterol Education Program Adult Treatment Panel III criteria. However, one should consider the possibility that any other available scheme to define the metabolic risk could be equally valid and produce different results. We did not account for the multiple testing inherent in examining 9 biomarkers, increasing the chance to introduce false positive findings. Because our sample represents mostly white individuals, the generalization of our findings to other ethnic/racial groups is uncertain. Although we selected a robust panel of inflammatory biomarkers, we recognized the limitation caused by missing information on biomarkers such as E-selectin, VCAM-1 or adiponectin. The strengths of the present study includes a large, community-based sample, a routine ascertainment of potential confounders and the availability of a robust set of inflammatory markers, using precise techniques to quantify their concentrations.

## Conclusions

Our study evaluated a panel of nine inflammatory biomarkers in a moderately large-sized cohort, and supports the hypothesis that metabolic syndrome as a construct generally is not more than the sum of its parts with respect to inflammation.

## Abbreviations

CRP = C-reactive protein BMI = Body mass index; HOMA-IR = Homeostasis model assessment insulin resistance index.

## Competing interests

This study was funded by the National Heart, Lung, and Blood Institute’s Framingham Heart Study N01-HC-25195; and by RO1-HL076784, RO1-HL064753, and R01-AG028321, the National Institutes of Health, National Center for Research Resources, General Clinical Research Centers Program, and by a Career Development Award from the American Diabetes Association (Dr. Meigs) Dr. Meigs was supported by NIDDK K24 DK080140. No other potential conflict of interest was reported. The authors declare that there is no duality of interest associated with this manuscript. An earlier version of this paper has been presented as an abstract at the 50^th^ Cardiovascular Disease Epidemiology and Prevention Conference 2010, San Francisco, USA.

## Author contributions

DD researched data, contributed to discussion and wrote the manuscript. MGL researched data, performed statistical analysis and reviewed/edited manuscript. RSV contributed to discussion and reviewed/edited manuscript. JFK supervised biomarker measurements, contributed to discussion and reviewed/edited manuscript. JDF contributed to discussion and reviewed/edited manuscript. JBM contributed to discussion and reviewed/edited manuscript. CSF conceived of the study, researched data, contributed to discussion and reviewed/edited manuscript. EJB conceived of the study, provided funding for the study, researched data, contributed to discussion and reviewed/edited manuscript. All authors read and approved the final manuscript

## Supplementary Material

Additional file 1 **Table S1.** Fold increments among the inflammatory biomarkers when comparing those with metabolic syndrome versus those without metabolic syndrome by BMI category. Click here for file

Additional file 2 **Table S2.** Fold increments among the inflammatory biomarkers when comparing those with metabolic syndrome versus those without metabolic syndrome by presence/absence of Insulin Resistance. Click here for file

Additional file 3 **Figure S1.** Geometric mean concentrations of C-reactive protein (CRP) by insulin resistance (IR) with/without metabolic syndrome (MetS) obtained from the multivariable-adjusted regression model with natural log(CRP) as dependent variable adjusting for age, sex, smoking, aspirin use and hormone replacement therapy. Whiskers extend to upper limits of two-sided 95% confidence intervals. IR defined as ≥ 75% of HOMA-IR. (p = 0.008 for interaction between metabolic syndrome and IR). Click here for file

Additional file 4 **Table S3.** Fold increments among the inflammatory biomarkers when comparing those with metabolic syndrome versus those without metabolic syndrome by Sex. Click here for file
