# Supplementary material for: Metabolic syndrome and inflammatory biomarkers: a community-based cross-sectional study at the Framingham Heart Study
Source: Diabetol Metab Syndr. 2012 Jun 20;4:28. doi: 10.1186/1758-5996-4-28 (PMC3547735; doi:10.1186/1758-5996-4-28)
Supplement: Additional file 1 — Table S1. Fold increments among the inflammatory biomarkers when comparing those with metabolic syndrome versus those without metabolic syndrome by BMI category. [file 1758-5996-4-28-S1.doc]

| **Supplementary Table 1.** Fold increments among the inflammatory biomarkers when comparing those with metabolic syndrome versus those without metabolic syndrome by BMI category | | | | |
| --- | --- | --- | --- | --- |
| **Body mass index:**  **Metabolic Syndrome Status:** | **<25 kg/m^2^**  **101 yes; 710 no** | **25-29.9 kg/m^2^**  **421 yes; 658 no** | **≥30 kg/m^2^**  **462 yes; 218 no** |  |
| **Biomarker** | Estimate (95% CI) | Estimate (95% CI) | Estimate (95% CI) | **Interaction**  P-value |
| **C-reactive protein** | 1.60 (1.31, 1.95) | 1.27 (1.13, 1.43) | 1.13 (0.96, 1.31) | **0.02** |
| **CD40Ligand** | 0.91 (0.70, 1.18) | 0.95 (0.82, 1.11) | 0.81 (0.66, 0.99) | 0.44 |
| **Intercellular adhesion molecule-1** | 1.06 (1.01, 1.11) | 1.03 (1.00,1.06) | 1.06 (1.02, 1.10) | 0.35 |
| **Interleukin-6** | 1.20 (1.05, 1.38) | 1.18 (1.08, 1.27) | 1.05 (0.94, 1.16) | 0.15 |
| **Monocyte chemoattractant -1** | 0.97 (0.90, 1.04) | 1.03 (0.99, 1.07) | 1.00 (0.95, 1.05) | 0.28 |
| **Osteoprotegerin** | 1.03 (0.97, 1.09) | 1.01 (0.97, 1.04) | 0.98 (0.94, 1.02) | 0.33 |
| **P-selectin** | 1.15 (1.07, 1.24) | 1.08 (1.03, 1.13) | 1.11 (1.05, 1.17) | 0.30 |
| **Tumor necrosis factor-alpha** | 1.17 (1.05, 1.31) | 1.00 (0.93, 1.07) | 1.13 (1.04, 1.24) | **0.01** |
| **Tumor necrosis factor receptor 2** | 1.07 (1.01, 1.13) | 1.06 (1.02, 1.09) | 1.03 (0.99, 1.08) | 0.53 |
| Adjusting for age, sex, smoking, aspirin use and hormone replacement therapy. | | | | |
