# Supplementary material for: Metabolic syndrome and inflammatory biomarkers: a community-based cross-sectional study at the Framingham Heart Study
Source: Diabetol Metab Syndr. 2012 Jun 20;4:28. doi: 10.1186/1758-5996-4-28 (PMC3547735; doi:10.1186/1758-5996-4-28)
Supplement: Additional file 2 — Table S2. Fold increments among the inflammatory biomarkers when comparing those with metabolic syndrome versus those without metabolic syndrome by presence/absence of Insulin Resistance. [file 1758-5996-4-28-S2.doc]

| **Supplementary Table 2.** Fold increments among the inflammatory biomarkers when comparing those with metabolic syndrome versus those without metabolic syndrome by presence / absence of Insulin Resistance | | | | |
| --- | --- | --- | --- | --- |
| **Insulin Resistance:**  **Metabolic Syndrome Status:** | **No**  **479 yes; 1363 no** | | **Yes**  **466 yes; 147 no** |  |
| **Biomarker** | **Estimate (95% CI)** | | **Estimate (95% CI)** | **Interaction**  **P-value** |
| **C-reactive protein** | | 1.67 (1.50, 1.86) | 1.25 (1.04, 1.51) | **0.008** |
| **CD40Ligand** | | 0.86 (0.75,0.98) | 0.97 (0.77, 1.22) | 0.36 |
| **Intercellular adhesion molecule-1** | | 1.02 (0.99,1.04) | 1.04 (1.00,1.09) | 0.43 |
| **Interleukin-6** | | 1.21 (1.13, 1.30) | 1.17 (1.03,1.32) | 0.62 |
| **Monocyte chemoattractant -1** | | 1.03 (0.99, 1.06) | 1.04 (0.98, 1.11) | 0.66 |
| **Osteoprotegerin** | | 0.99 (0.96,1.01) | 1.02 (0.97,1.08) | 0.20 |
| **P-selectin** | | 1.11 (1.07,1.15) | 1.05 (0.99,1.13) | 0.22 |
| **Tumor necrosis factor-alpha** | | 1.07 (1.01, 1.13) | 0.98 (0.88, 1.08) | 0.13 |
| **Tumor necrosis factor receptor 2** | | 1.05 (1.02, 1.08) | 1.03 (0.98,1.08) | 0.59 |
| Adjusted for age, sex, smoking, aspirin use, hormone replacement therapy. IR defined as ≥ 75^th^ percentile of values of HOMA-IR. | | | | |
