# Supplementary material for: Metabolic syndrome and inflammatory biomarkers: a community-based cross-sectional study at the Framingham Heart Study
Source: Diabetol Metab Syndr. 2012 Jun 20;4:28. doi: 10.1186/1758-5996-4-28 (PMC3547735; doi:10.1186/1758-5996-4-28)
Supplement: Additional file 3 — Figure S1. Geometric mean concentrations of C-reactive protein (CRP) by insulin resistance (IR) with/without metabolic syndrome (MetS) obtained from the multivariable-adjusted regression model with natural log(CRP) as dependent variable adjusting for age, sex, smoking, aspirin use and hormone replacement therapy. Whiskers extend to upper limits of two-sided 95% confidence intervals. IR defined as ≥ 75% of HOMA-IR. (p = 0.008 for interaction between metabolic syndrome and IR). [file 1758-5996-4-28-S3.doc]

| **Supplementary Table 3.** Fold increments among the inflammatory biomarkers when comparing those with metabolic syndrome versus those without metabolic syndrome by Sex | | | | |
| --- | --- | --- | --- | --- |
| **Sex:**  **Metabolic Syndrome Status** | **Men**  **476 yes; 628 no** | | **Women**  **508 yes; 958 no** |  |
| **Biomarker** | **Estimate (95%CI)** | | **Estimate (95% CI)** | **Interaction**  **P-value** |
| **C-reactive protein** | | 1.47 (1.30, 1.65) | 2.17 (1.95, 2.43) | <0.0001 |
| **CD40Ligand** | | 0.91 (0.78, 1.05) | 0.87 (0.76, 0.99) | 0.69 |
| **Intercellular adhesion molecule-1** | | 1.05 (1.02, 1.08) | 1.05 (1.03,1.08) | 0.95 |
| **Interleukin-6** | | 1.25 (1.15, 1.35) | 1.32 (1.23,1.42) | 0.28 |
| **Monocyte chemoattractant -1** | | 1.05 (1.01, 1.09) | 1.02 (0.99,1.06) | 0.40 |
| **Osteoprotegerin** | | 0.98 (0.95,1.01) | 1.02 (0.99, 1.05) | 0.11 |
| **P-selectin** | | 1.09 (1.04,1.13) | 1.14 (1.09, 1.18) | 0.12 |
| **Tumor necrosis factor-alpha** | | 1.05 (0.98, 1.12) | 1.14 (1.07, 1.21) | 0.08 |
| **Tumor necrosis factor receptor 2** | | 1.05 (1.01,1.08) | 1.12 (1.09,1.15) | 0.002 |
| Adjusted for age, smoking, aspirin use, hormone replacement therapy. | | | | |
