# Supplementary figures and images for: Metabolic syndrome and inflammatory biomarkers: a community-based cross-sectional study at the Framingham Heart Study
Source: Diabetol Metab Syndr. 2012 Jun 20;4:28. doi: 10.1186/1758-5996-4-28 (PMC3547735; doi:10.1186/1758-5996-4-28)

**Supplementary Figure 1**


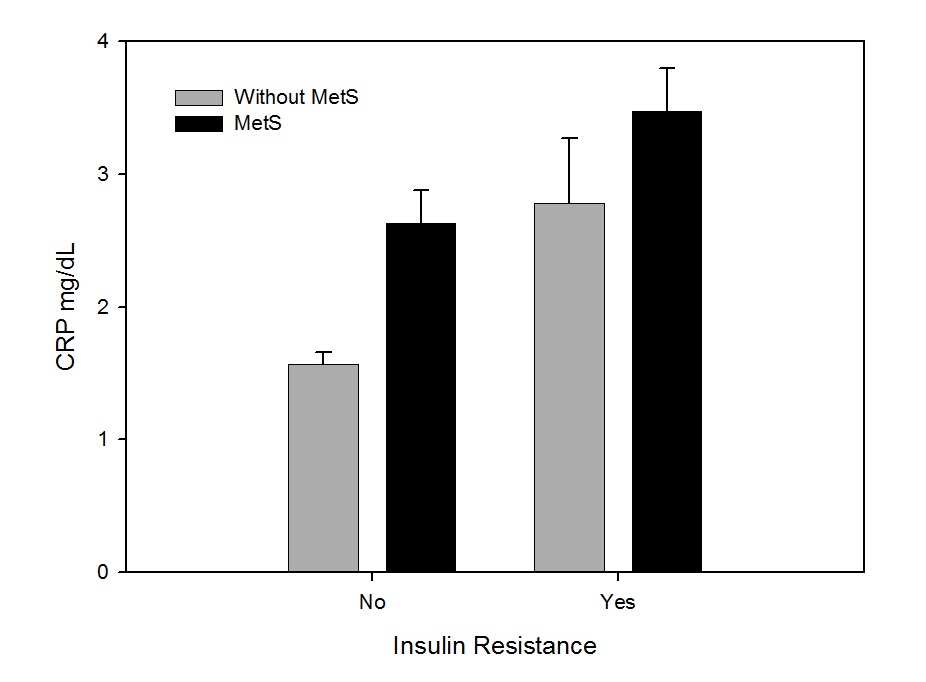

Supplement: Additional file 4 — Table S3. Fold increments among the inflammatory biomarkers when comparing those with metabolic syndrome versus those without metabolic syndrome by Sex. [file 1758-5996-4-28-S4.doc]
